# Impact of Fatty Acid Supplementation on Cognitive Performance among United States (US) Military Officers: The Ranger Resilience and Improved Performance on Phospholipid-Bound Omega-3’s (RRIPP-3) Study

**DOI:** 10.3390/nu13061854

**Published:** 2021-05-29

**Authors:** Bernadette P. Marriott, Travis H. Turner, Joseph R. Hibbeln, Jill C. Newman, Marcie Pregulman, Angela M. Malek, Robert J. Malcolm, Gregory A. Burbelo, Jeffrey W. Wismann

**Affiliations:** 1 Division of Gastroenterology and Hepatology, Department of Medicine, College of Medicine, Medical University of South Carolina, 114 Doughty Street, Charleston, SC 29425, USA; newmanji@musc.edu; 2 Department of Psychiatry and Behavioral Sciences, College of Medicine, Medical University of South Carolina, 67 President Street, Charleston, SC 29425, USA; malcolmr@musc.edu; 3 Department of Neurology, College of Medicine, Medical University of South Carolina, 96 Jonathan Lucas Street, Suite 301 CSB, Charleston, SC 29425, USA; turnertr@musc.edu; 4 Psychiatry and Behavioral Health, Barton Health, 2209 Second Avenue, South Lake Tahoe, CA 96150, USA; jhibbeln@bartonhealth.org; 5 Division of Nephrology, Department of Medicine, College of Medicine, Medical University of South Carolina, 96 Jonathan Lucas Street, CSB, HE814, MSC629, Charleston, SC 29425, USA; pregulma@musc.edu; 6 Department of Public Health Sciences, College of Medicine, Medical University of South Carolina, 135 Cannon Street, Suite 303C, Charleston, SC 29425, USA; malek@musc.edu; 7 LTC, Infantry, United States Army, 432 Lee Road.2069, Smiths Station, AL 36877, USA; gburbelo@gmail.com; 8 Major, US Army Battalion Operations Officer, 4-23 Infantry Regiment, Joint Base Lewis-McChord, WA 98433, USA; Jeffrey.w.wismann.mil@mail.mil

**Keywords:** omega-3 fatty acids, cognitive performance, military officers, randomized controlled trial, United States, krill oil

## Abstract

Studies have assessed omega-3 fatty acids and cognitive decline among older adults and cognitive development among children, although less is known about cognitive or neurological effects among young adults. We examined whether omega-3 supplementation from krill oil could improve cognition and resilience among young military officers compared to a control. This double-blind, placebo-controlled trial enrolled 555 officers (mean age 23.4 ± 2.8, 98.6% male) entering the United States (US) Army Infantry Basic Officer Leaders Course (IBOLC) with the intention to complete the US Ranger Course. Volunteer participants consumed eight dietary supplements daily of krill oil containing 2.3 g omega-3 or control (macadamia nut oil) over an approximate 20-week period. Cognitive functioning, resilience, and mood were assessed during a well-rested period at approximately 14 weeks and after a battlefield simulation at 16 weeks. Blood spot samples were collected to monitor compliance and dietary intake was assessed. All hypotheses were tested using both ‘Intention to Treat’ (ITT) and ‘As Per Protocol’ (APP) approaches. Of the 555 randomized individuals, 245 (44.1%) completed the study. No statistically significant group-by-time interactions indicating treatment effect were found on any outcomes. Poor compliance was indicated by lower than expected omega-3 elevations in the treatment group, and may have contributed to a failure to detect a response.

## 1. Introduction

Polyunsaturated fatty acids (PUFAs) include omega-3 highly unsaturated fatty acids: (*n − 3*: alpha lenolenic acid [ALA]), eicosapentaenoic acid (EPA), docosahexaenoic acid (DHA)*,* and omega-6 (*n − 6*) fatty acids: linoleic acid (LA) and arachidonic acid (ARA). PUFAs are concentrated in neural tissues and are deemed essential for neural and neurotransmitter function among adults [1,2,3] and for neurodevelopment among children [4,5]. Adequate *n − 3* and *n − 6* fatty acids cannot be synthesized in the body and must be obtained from the diet. The primary sources for EPA and DHA are fatty fish such as salmon and herring, and dietary supplements (DSs) such as cod liver oil; ALA is obtained from plant sources such as walnuts and flax seeds [6]. The main sources of *n − 6* fatty acids, primarily consumed as LA, are plant oils such as canola, corn, and soybean oil [6,7].

Omega-3 fatty acids have been studied in both observational and randomized controlled trials (RCTs) for their potential importance in terms of emotional states, mental health, and cognitive function cf., [8,9]. Cognitive research with *n − 3* fatty acids has focused on development among children and cognitive decline among older populations [10]. A recent summary has concluded that relatively few studies have concentrated on fatty acids and cognitive performance among healthy young adults [3].

The purpose of the Ranger Resilience and Improved Performance on Phospholipid-bound Omega-3’s (RRIPP-3) study was to determine whether supplementation with *n − 3* fatty acids could enhance resilience to stress in healthy young adults as demonstrated by improving the results on appropriate cognitive tests compared to control. The cognitive assessments were selected as a representative of key performance elements during the United States (US) Army Infantry Basic Officer Leaders Course (IBOLC). For the US Army Infantry, IBOLC, located in Fort Benning, Georgia, is the initial training station for officers who have graduated from the US Military Academy (USMA), completed Officer Candidate School (OCS) or Reserve Officer Training Corps (ROTC). Immediately after graduation from the 17–19 week-long IBOLC training, the majority of IBOLC participants volunteer to attend the Army’s Ranger Course. The Ranger Course is the US Army’s premier small unit tactics and leadership school and is eight weeks in duration. The course focuses on developing and assessing students’ ability to lead under extreme physically and mentally demanding conditions. The RRIPP-3 study employed cognitive tests designed for healthy adults and administered these tests at baseline, during a rested state at 14 weeks, and immediately following an intense 3-day combat simulation challenge at about 16 weeks, a program time point when IBOLC training is most stressful, and with an additional study testing before and after the Ranger Course. For this study, *n − 3* fatty acids were supplied by krill oil. Fatty acids in krill oil differ in their physiochemical properties from fatty acids in fish oil in that DHA is primarily phospholipid-bound, which enhances transport into the brain, whereas in fish and fish oil, EPA and DHA are primarily bound to triglycerides (TG) [11]. The main transporter for DHA across the blood–brain barrier is lyso-phosphatidylycholine [12].

## 2. Materials and Methods

Details of the methods for the RRIPP-3 study have been provided elsewhere [13]. Here we provide the key elements of the study design.

### 2.1. Eligibility and Recruitment

**Figure 1:**
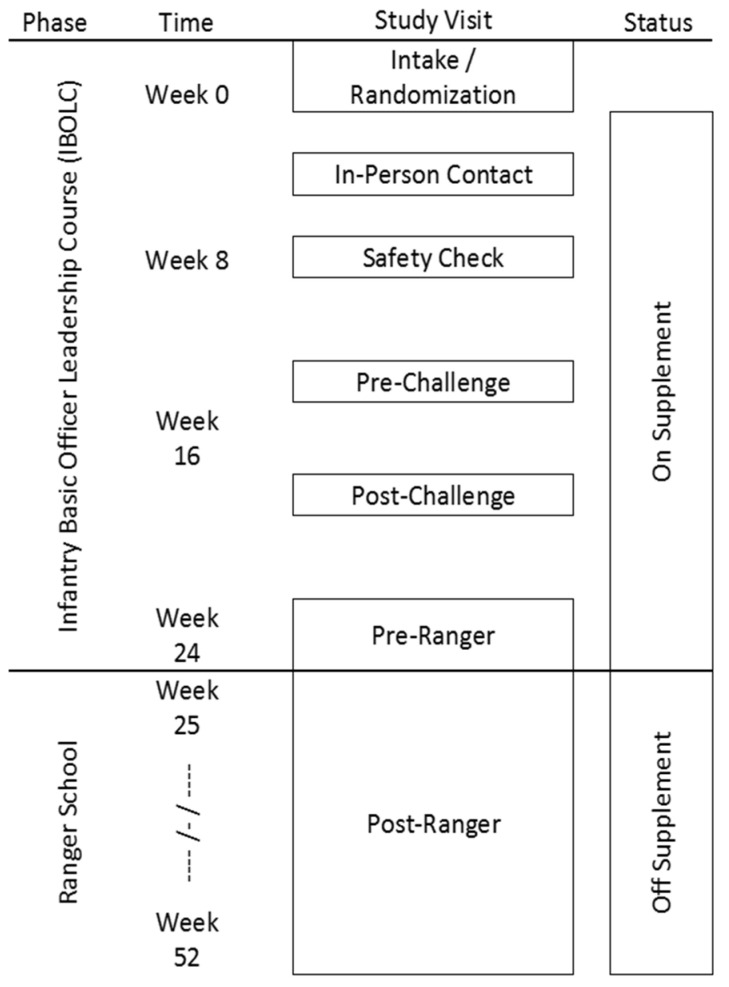
RRIPP-3 study flow diagram illustrating the study visits for the participants while taking the experimental or control supplements.

New participants are enrolled into the US Army IBOLC at the beginning of each month for 11 months each year. IBOLC and the Ranger Course provide a rigorous and physically demanding program coupled with specific training in small unit tactics in challenging environments, stressing situation awareness, critical analysis, and decision-making while under acute stress. In the final two weeks of training, the skills learned during IBOLC are tested in a culminating challenge exercise (Leader Forge) which, during the RRIPP-3 study, included nine days of near-continuous field training designed to simulate battlefield conditions and provide a scenario where Army trainers can evaluate junior officers’ ability to perform in combat [13]. The RRIPP-3 study included cognitive tests prior to and after Leader Forge as part of its study design to test cognitive performance under stress (Pre- and Post-Challenge in Figure 1).

All healthy US Army IBOLC students, typically male, age 20–35 years, who were planning to enter Ranger School after IBOLC were eligible to participate in RRIPP-3 if they had no previous injuries or existing physical limitations that would prevent their successful performance, no known allergies to fish or nuts, no history of non-febrile seizures, no autoimmune diseases, no diagnosis of Type I or Type II diabetes or coronary heart disease, and presently had no infections or fevers of an unknown origin. Eligible participants were also required to have not typically consumed seafood three or more times per week within the last three months and to have had no consumption of hypoglycemic agents or regular use of omega-3-containing supplements within the last three months. Both men and women of all racial and ethnic groups were eligible to participate. International students with previous military training that differed from the US Army and officers who were enrolled in the US National Guard and were subject to unanticipated service-based departure, were excluded from recruitment. Study participants were asked to agree to stop consuming dietary supplements that conflicted with the study goals, to avoid consuming macadamia nuts, to not increase omega-3 consumption through their diet, and to consume the DSs provided through the study.

Officers were recruited during the first assembly on the initial day of each of the 12 IBOLC classes from August 2016 through to November 2017. Interested officers signed up for a screening/enrollment visit and were asked to bring all DSs they typically consumed with them to the enrollment meeting, which took place within 72 h of screening. At the enrollment meeting, two short videos were shown to ensure that potential participants received the same information about the study expectations, the informed consent, and the Health Insurance Portability and Accountability Act (HIPAA) process. After seeing the informational videos at the screening visit, the officers had the opportunity to ask questions and decide about study participation. Study coordinators also explained and reviewed the inclusion/exclusion information. At that time, study coordinators also reviewed the DSs that each participant had brought with them to the baseline enrollment visit and received a verbal commitment from the study participants to comply with the study protocol and to cease consuming any DSs that conflicted with the study. Study participants then signed the informed consent and HIPAA documents, participated in the baseline enrollment study series of assessments, were randomized using a blinded code system generated and known only by the lead study statistician and the medical monitor, and received their first 8-week supply of study DSs.

### 2.2. Intervention Products

Experimental and control groups were provided with identical-appearing black gelatin capsules in 8-capsule, daily dose, blister packs. Both the experimental and control group supplements were custom manufactured to look and smell the same. However, if the capsules were crushed or broken, the experimental capsules presented a fishy odor. Participants were asked to consume all 8 capsules per day. Blister packs were provided in boxes that contained one week’s supply and participants received an 8-week supply during each study visit. DSs were given to study participants only during IBOLC and not during the Ranger Course.

The experimental group received DS capsules containing a relatively high amount of concentrated krill oil extracted from *Euphausia superba* yielding ≈2.3 g/d omega-3s with a EPA and DHA ratio of approximately 2:1 (See Table 1). The control group received identically colored and sized capsules containing macadamia nut oil containing between 53% and 67% oleic acid and 16% and 24% palmitoleic acid, determined by independent analysis See Table 2). Macademia nuts are rarely consumed by the study population. In addition, macadamia nut oil is high in 16:1n − 7 and it was hoped that this biomarker could be used to assess compliance in the placebo group. The experimental and control DS were produced and supplied by Aker BioMarine Antarctic, AS, Fjordalleen 16, 0115 Oslo, Norway. Additional information about the DS products can be found in the detailed methods publication [13].

### 2.3. Study Visits, Hypotheses, and Cognitive Tests

RRIPP-3 participants were expected to meet with study coordinators for five study visits during which cognitive assessments were conducted across the 17-week duration of IBOLC (during 2016 and 2017) or the 19-week duration of IBOLC (during 2018) (Phase I): at baseline, Pre-Challenge (Leader Forge), Post-Challenge, Pre-Ranger, and after participation in the Ranger Course. A safety check visit was held at week 8 to verbally evaluate study participants for any side effects of the experimental or control supplements. Figure 1 illustrates the study flow.

RRIPP-3 sought to test four hypotheses and the cognitive tests that were used were selected to isolate the cognitive functioning that was tested in these hypotheses. Specifically, we hypothesized that dietary supplementation with krill oil concentrate would:

1. Improve attention, cognitive processing speed, and executive control as measured by performance on computerized adaptions of the Stroop Color-Word Inhibition test [14] and Symbol-Digit Modality Test (SDMT) [15], from baseline to mid-points (Pre- and Post-Challenge; See Figure 1) and at the conclusion of IBOLC training, as compared to the control.
2. Enhance psychological and physiological resiliency, as measured by responses to the Connor-Davidson Resilience Scale [16] and the Patient-Reported Outcomes Measurement Information System (PROMIS) [17]) from baseline to mid-points (Pre- and Post-Challenge) and at the conclusion of IBOLC training, as compared to control.
3. Improve real-world visuospatial planning, as measured by performance in Land Navigation tests administered by the US Army IBOLC training program between weeks 6 and 8. Land navigation tests the ability of candidates to navigate from one point to another using a map and compass while equipped with their individual combat gear.
4. Improve real-world visual psychomotor control, as measured by performance in the Marksmanship tests administered by the US Army IBOLC training program between weeks 2 and 4 compared to control. Controlled and accurate use of firearms is essential for the Army officer. In addition to understanding the physics and mathematical adjustment for environmental conditions, marksmanship requires proper body mechanics, focus, breathing control, and visual psychomotor skills.

RRIPP-3 also recorded additional facets of cognitive functioning and psychological states at each study visit as secondary outcomes: working memory (Figural Continuous Paired Associates Test [18]), reasoning (Grammatical Reasoning Test [19]), risky decision making and risk-taking behavior (Balloon Analogue Risk Task [20]), visual attention (Four-Choice Visual Reaction Time Test [21]), dispositional and trait anxiety (Spielberger State/Trait Anxiety Inventory [22]), stress (Peritraumatic Distress Inventory [23]), mood state (Profile of Mood States-Bipolar [24]), and narcissism (Narcissistic Personality Inventory [25]).

RRIPP-3 study participation required potential IBOLC study participants to intend to participate in the Ranger Course. Thus, successful graduation from the Ranger Course was included as a secondary outcome measure, which the fatty acid intervention was hypothesized to affect, as well as the stress related to Ranger School failure. The Peritraumatic Distress Inventory [23] was administered during the study visit that occurred immediately after completion of the Ranger Course as a measure of exposure to a significant traumatic event.

### 2.4. Dietary Assessment and Study Protocol Compliance

Dietary intake was assessed at baseline and at the end of the IBOLC visit prior to entry into the Ranger Course using the US Department of Agriculture (USDA) Automated Multiple Pass Method (AMPM) 24-h, interviewer-based dietary recall [26] and the Diet History Questionnaire (DHQ) III, which is a 30-day food frequency questionnaire [27]. DS use was recorded to generate an estimate of total usual nutrient intake [28] and to ensure that the intervention was not compromised. Participants also reported the number of days in the past 30 days in which they engaged in moderate or vigorous physical activity during their leisure time. Examples of moderate physical activity were given as walking briskly, mowing the lawn, dancing, swimming or bicycling, with examples of vigorous activities listed as jogging, chopping wood, swimming continuous laps or bicycling uphill [29].

Blood spot samples of non-fasting capillary blood were obtained on BHT/EDTA-impregnated filter paper by finger prick at baseline and at all visits for determination of participant study protocol compliance through analyses of fatty acids (experimental group) and oleic and palmitoleic acids (control group). A high flow 18G safety lancet (Assure Haemolance Plus, Arkay Inc., Edina, MN, USA) was used to obtain the samples, which were air dried for 3 h at room temperature, stored in containers under refrigeration and shipped to the US National Institutes of Health (NIH) where they were analyzed for fatty acid composition by the method of Lin et al. [30] in the laboratory of a co-investigator (JRH). Upon analysis, fatty acid blood values for one full group of recruits was extraordinarily high. This full group (*n* = 35) was excluded from statistical analyses because the blood samples were deemed to be contaminated prior to laboratory analyses.

### 2.5. Statistical Design and Tests

A stratified block design was used to randomize the study volunteers to insure that the two treatment groups would be balanced by commissioning source (Army ROTC, USMA, or OCS) and post-graduate destination to either an Armored Brigade Combat Team (ABCT), a Stryker Brigade Combat Team (SBCT), or an Infantry Brigade Combat Team (IBCT), because prior analyses by the US Army had indicated that these factors were associated with success in Ranger School. The block size and assignments (experimental or control group) were unknown to the investigators and were only known by the lead study statistician (JCN) and the medical monitor (RJM). The assignment to an experimental or a control group was randomly arrayed and balanced within each block by the RRIPP-3 statistician. Based on power calculations as detailed in our previously published methods paper [13], a total analytic sample size of 268 participants was required.

### 2.6. D. Statistical Tests

All hypotheses were tested using ITT and APP approaches. In the ‘intent to treat’ analyses, all participants randomized to the experimental treatment group were compared to all participants randomized to the control group. In the ‘As Per Protocol’ analyses, we classified participants based on perceived compliance with supplementation, based on blood spot sample analyses. All participants with a 25% increase in omega-3 (20:5n − 3 and 22:6n − 3) as measured by blood spot samples from baseline to the either Pre-Challenge or Post-Challenge (approximately weeks 15 and 17) study, visits were deemed compliant with the study protocol. Upon initial analysis, the levels of oleic and palmitoleic acid in the baseline samples resulting from the participants’ diet alone were very high and there was no expectation that the macadamia nut oil supplements would result in a measurable increase in the samples over the study period. A reverse compliance approach was applied for the control group, and only those participants in the control group whose omega-3 blood levels did not increase by 25% were used for the ‘intent to treat’ analyses on outcome measurements as compliers. Details of the data acquisition and analytic approach are provided elsewhere [13].

Previous studies have shown that a proportion of participants in RCTs involving cognitive outcomes do not provide an adequate effort on testing, and inclusion of invalid cognitive testing data can produce spurious findings of change with treatment or obscure actual experimental effects [31]. Accordingly, data from cognitive tests at all assessments were screened to exclude cases where performance fell at chance-level guessing (binomial probability <0.80 for successes) or represented extreme outlier scores (>3 interquartile ranges below first quartile). Of the primary outcome measures, for the Stroop test, 39 cases (7%) were excluded at baseline, 5 cases were excluded at Pre-challenge (1%), and 19 cases were excluded at post-challenge (6%). No poor effort cases were identified for any participants on any administration of the Symbol Digit Modality Test (SDMT).

To generate the analysis data set for each primary and secondary outcome, participants with complete data for all three time points (Baseline, Pre-Challenge and Post-Challenge) were included, as well as participants with Baseline and Post-Challenge data. For participants with Baseline and Post-Challenge data only, a Pre-Challenge value was imputed using a Markov Chain Monte Carlo algorithm for multiple imputation with ten replications under Missing at Random (MAR) assumptions. Using this method allowed an analysis sample size that was above the estimated sample size needed at 80% power to detect an effect. Sensitivity analyses were run for each outcome with and without imputation. The results using each method were consistent.

Descriptive statistics (e.g., means, standard deviations, medians, percentages) were used to characterize cognitive function in the 2 treatment groups. Student’s *t*-tests, chi-square tests, and Wilcoxon Rank Sum non-parametric tests were used to compare baseline characteristics between groups.

A mixed modeling approach for repeated measures was used to assess differences by treatment over the three IBOLC time points. A time by treatment interaction was used for this assessment, implementing both the fixed and random effects in each model. Least square means, differences in least square means and *p*-values were reported for each primary outcome. Before analysis, a 99% winsorizing Macro was applied to the Total Score dataset for the Spielberger Trait Anxiety Inventory to correct for distribution outliers. All analyses were conducted using SAS version 9.4 (SAS Institute, Cary, NC, USA) and a *p*-value < 0.05 was considered statistically significant.

The study was reviewed and approved by the Medical University of South Carolina (MUSC) Institutional Review Board and Human Subject Protection Program (IRB) and the United States Army Center for Initial Military Training Research Review Group, which is responsible for research at Fort Benning, GA. RRIPP-3 is registered with ClinicalTrials.gov as study NCT02908932 with unique protocol identifier Pro00051532.

## 3. Results

### 3.1. Study Participants

**Figure 2:**
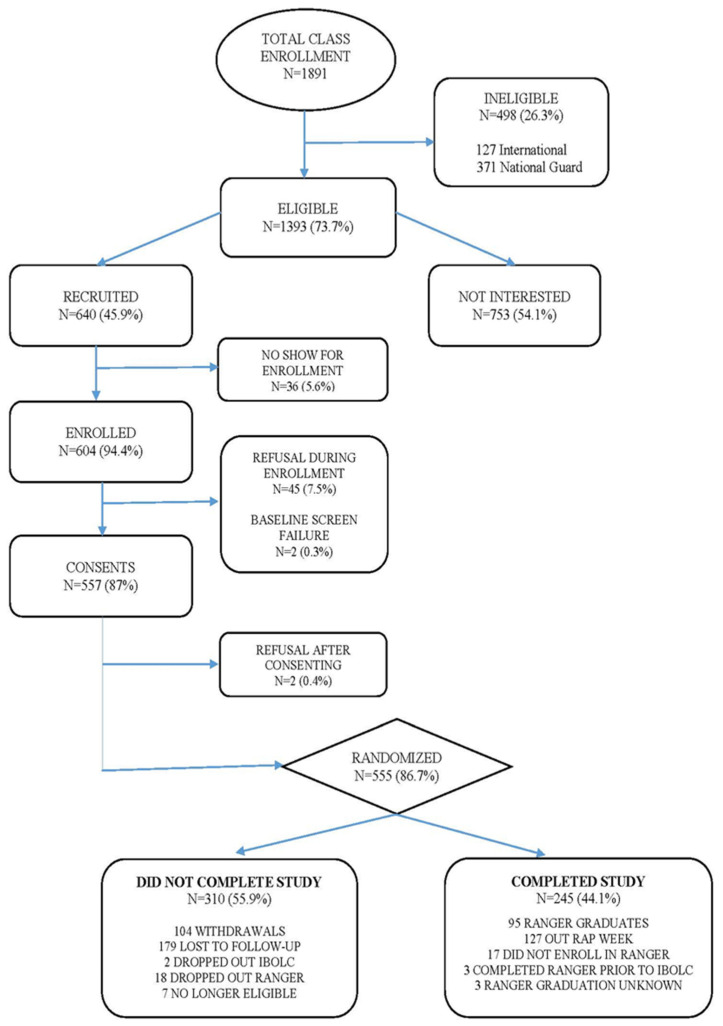
RRIPP-3 CONSORT Diagram (note: RAP week is particularly challenging first week of Ranger training).

The recruitment, enrollment and participant completion of the RRIPP-3 study are shown in the Consolidated Standards of Reporting Trials (CONSORT) diagram (Figure 2). RRIPP-3 participants were recruited during the initiation of 12 IBOLC classes from which 1891 were enrolled. Of those, 498 were not eligible for the RRIPP-3 study because they were international students with previous training that was different from the US Army or they were enrolled in the US National Guard and subject to unanticipated service-based departure from IBOLC. After enrollment was completed, 555 (86.7%) of the eligible individuals were randomized with 274 (49.7%) in the experimental group and 279 (50.5%) in the control group. Of these 555 participants, 245 individuals (44.1%) completed the study: 130 from the experimental group and 115 from the control group. Those 179 participants who did not continue the study, upon questioning, primarily reported that they kept forgetting to take their DSs and therefore decided to drop out. Fifteen adverse events were reported during the study (7 in the experimental group and 8 in the control group). No adverse events were considered serious.

The final randomized study population was young, predominantly non-Hispanic White, and male with a Bachelor’s degree and were never married. The randomization design balanced for commissioning source and post-graduation destination, which led to similar groups in demographic factors with no statistically significant differences between the experimental and control groups for any characteristics (See Table 3 and Appendix A, Table A1) Baseline health and lifestyle characteristics also did not differ significantly between the randomized groups, with over 90% of the participants stating they were in good or excellent health, 41% reporting having never smoked more than 100 cigarettes, 42% stating they had never used chewing tobacco, and over 65% stating that they engaged in moderate or vigorous physical activity more than five days per week. See Table A1 in Appendix A.

### 3.2. Nutrient Intake

Dietary Intake was assessed to determine the general nutrient intake of the study participants at baseline and to determine if there was any difference between the control and experimental groups in consumption of omega-3 fatty acids prior to the study. In our study sample, 54.6% reported taking DSs at baseline, which was similar to the general population as reported by the Center for Disease Control and Prevention (CDC) based on the National Health and Nutrition Examination Survey (NHANES), which was conducted at the time of the RRIPP3 study in 2017–2018, which shows that 50.8% of men over 20 years of age use DSs [32]. Table 4 shows the estimated nutrient intake from diet and DSs for the total study population at baseline in comparison with the Military Dietary Reference Intakes (MDRIs). The MDRIs establish standards intended to meet the nutrient requirements of warfighters and are outlined in Army Regulation 40–25, OPNAVINST 101 10.1/MCO10110.49 AFI 44-14 [33]. The MDRIs are based upon the Dietary Reference Intakes (DRIs), which are developed by the Food and Nutrition Board, US National Academy of Sciences and represent the current knowledge of the nutrient needs of a healthy population [34]. In addition, the MDRIs incorporate the most current understanding of the military population and the nutrient demands of their activities [33].

In our study population, the nutrient intake from the diet alone for 20 of the 26 nutrients for which there are MDRIs, was higher than the MDRI. When total dietary intake was considered by adding the DS nutrients, the total intake was much higher than the MDRI for these nutrients. Surprisingly, energy and carbohydrate intake from the diet plus DSs was less than the MDRI. Vitamins D and E, which have been reported as short-fall nutrients in the diets of military personnel in other studies [35], did not exceed the MDRI recommendations in this study with the addition of these nutrients from DSs. At baseline, there was no statistically significant difference between the experimental and control groups for intake of omega-3 highly unsaturated fatty acids: (*n − 3:* ALA, EPA, DHA or overall between experimental and control groups in total dietary intake for any nutrient (*p* = 0.5199, 0.2208, 0.6694). No MDRI or Food and Nutrition Board DRI recommendations are available for EPA and DHA [29].

### 3.3. Intent-to-Treat Analysis

**Hypothesis** **1.** *Attention, Processing Speed, and Executive Control.*

**Figure 3:**
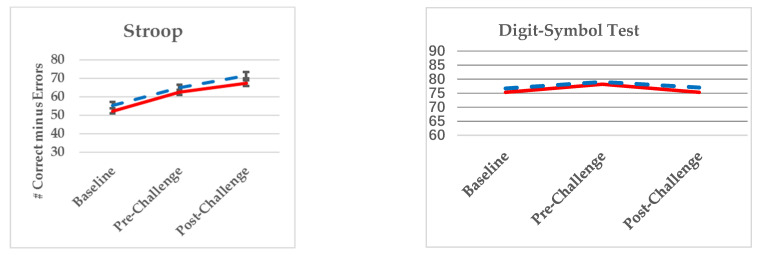
Performances on the primary cognitive outcomes by group across visits.

Figure 3 presents least square means (+/−SE) for performances across visits for the two primary cognitive assessments, the Stroop Color-Word Inhibition test and the SDMT, where the experimental group is shown in blue and the control group in red. No statistically significant differences by treatment group were observed for either measure; however, statistically significant main effects for time on the Stroop (*p* = <0.0001) and SDMT (*p* = 0.0302) were observed, suggesting an improvement in follow-up visits relative to baseline for the Stroop and a spike in Digit Symbol Test Scores at Pre-Challenge. The interaction of Group × Time, which indicate treatment effect, was not significant for the Stroop (*p* = 0.4683) or SDMT (*p* = 0.9121).

**Hypothesis** **2.** *Resilience.*

**Figure 4:**
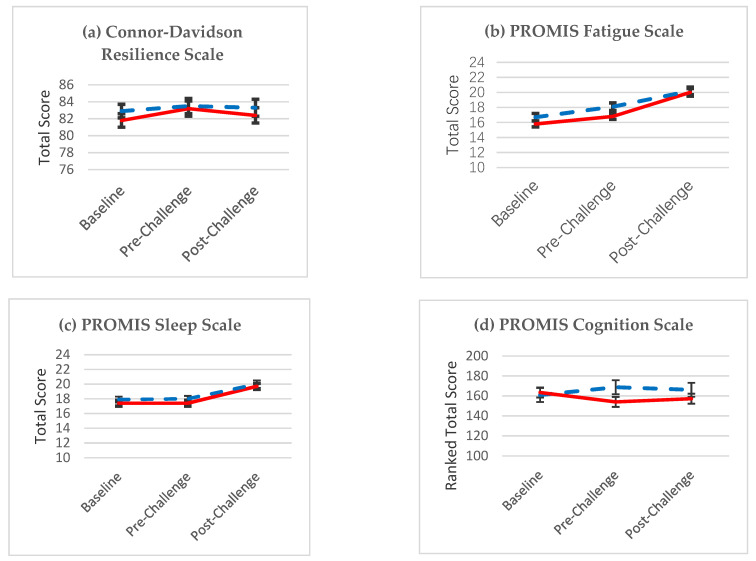
Least square means (+/−SE) for the four primary resilience outcomes: (**a**) the Connor-Davidson Resilience Scale and the three Patient-Reported Outcomes Measurement Information System (PROMIS) Scales: (**b**) Fatigue; (**c**) Sleep; and (**d**) Cognition, where the experimental group is shown in blue with the control group in red.

Figure 4 presents least square means (+/−SE) for the four primary resilience outcomes: the Connor-Davidson Resilience Scale [16] and the three PROMIS scales [17], with the experimental group shown in blue and the control group in red. No statistically significant group differences were observed. However, a statistically significant main effect of time was observed for the PROMIS Fatigue and Sleep-Related Cognitive Impairment Scales, indicating greater impairment at follow-up relative to baseline visits. The interaction of Group × Time, which would indicate treatment effect, was not significant for the Connor-Davidson Resilience Scale (*p* = 0.7287), PROMIS Fatigue Scale (*p* = 0.1281), PROMIS Sleep Related Impairment Scale *(p* = 0.6878), or the PROMIS Applied Cognition Scale (*p* = 0.7789).

**Hypothesis** **3.** *Real-World Visuospatial Planning.*

**Hypothesis** **4.** *Real-World Visuomotor Control.*

The IBOLC administration and operations changed many times during the course of the study. As a result, quantitative performance metrics on land navigation and marksmanship were no longer systematically collected over the course of the study. Specifically, the scoring of land navigation and marksmanship was revised several times during the RRIPP-3 study and resulted in unequal scoring measures across the study participants. Therefore, Hypotheses 3 and 4 could not be tested adequately.

### 3.4. Secondary Outcomes

**Figure 5:**
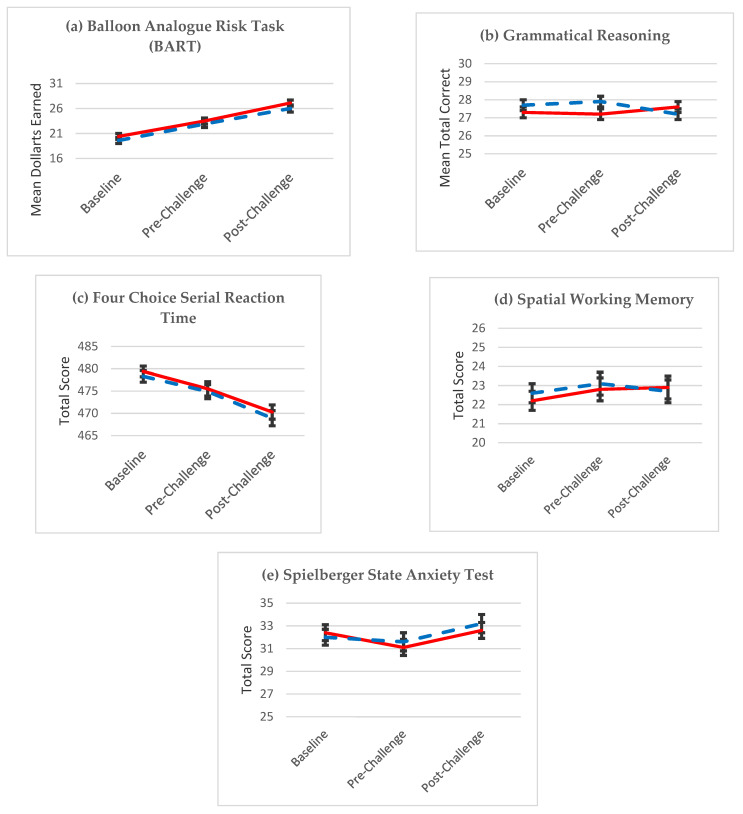
Least square means (±SE) by treatment at three time points during IBOLC for the (**a**) Balloon Analogue Risk Task (BART); (**b**) Grammatical Reasoning test; (**c**) Four Choice Serial Reaction Time test; (**d**) Spatial Working Memory Test; and (**e**) Spielberger State Anxiety Test, where the experimental group is shown in blue with the control group in red.

**Figure 6:**
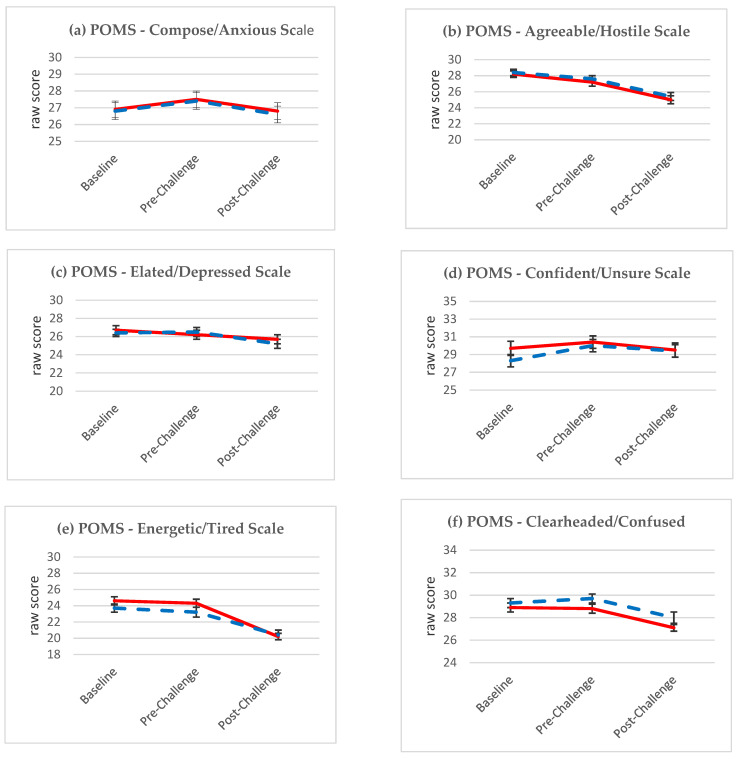
Least square means (±SE) by treatment at three time points during IBOLC for the Profiles of Mood State, bi-polar form (POMS) for six scales (**a**–**f**) at Baseline, Pre-Challenge and Post-Challenge during IBOLC, with the experimental group shown in blue and the control group in red.

The results of the secondary cognitive outcomes with the intent-to-treat analysis are included in Figure 5 and Figure 6. In Figure 5, for each outcome of the Balloon Analogue Task (BART) (*p* = 0.3930), the Grammatical Reasoning Test (*p* = 0.1119), the Four Choice Serial Reaction Time Test (*p* = 0.7068), the Spatial Working Memory Test (*p* = 0.5095), and the Spielberger State Anxiety Inventory (*p* = 0.8611), no statistically significant differences between the main effects of groups (omega-3 supplement versus control) and no significant interaction between group and time were seen.

In Figure 6, the results of the Profiles of Mood State, bi-polar form (POMS), where higher numbers in the assessment indicate a more favorable disposition, are illustrated for each of the six sub-scales: (a) Composed/Anxious (*p* = 0.8439); (b) Agreeable/Hostile (*p* = 0.8190); (c) Elated/Depressed (*p* = 0.2103); (d) Confident/Unsure (*p* = 0.6654); (e) Energetic/Tired (*p* = 0.0975); and (f) Clearheaded/Confused (*p* = 0.9949). There were no statistically significant main effects of time or group and the interaction between time and group was not statistically significant for any of the subscales. In three of the POMS models, a statistically significant main effect of time was demonstrated in the analysis with a statistically significant improvement in mood from baseline to before or after challenge tests in both groups (Energetic/Tired (time: *p* < 0.0001); Clearheaded/Confused (time: *p* < 0.0001); Agreeable/Hostile (time: *p* < 0.0001).

### 3.5. Study Participants Success in the Ranger Course

Since all individuals who were admitted to RRIPP-3 were required to have the goal of participation in the Ranger School, the effect of the study supplements on overall success in Ranger School was evaluated by treatment group. Of the study participants, 222 RRIPP-3 participants went to Ranger School after IBOLC. Of the 222 who entered Ranger School, 95 (42.8%) graduated from Ranger School with 48 (42.1%) participating in the experimental group and 47 (43.5%) having received the control supplements; no statistically significant difference was seen between the groups (*p* = 0.8319). RRIPP-3 supplements were discontinued at the beginning of Ranger School as required by the US Army.

### 3.6. As Per Protocol (AAP) Analysis

The RRIPP-3 ITT analysis assumed that participants randomly assigned to control (macadamia nut oil) and those assigned to the experimental (krill oil) would consume the DSs allocated to them throughout the study. Compliance in a randomized controlled trial is notoriously challenging and even more so in a military training setting where the participants are confronted daily with new physical and mental tasks. As a result, RRIPP-3’s study design included blood spot samples at each study visit to assess blood lipid profiles to reflect the experimental and control products as part of an APP statistical data analysis. These blood values are presented in Appendix B, Table A2. The study protocol identified an increase of ≥25% from baseline omega-3 blood levels to either pre- or post-challenge time points as indicative of compliance. Due to the variable ability of participants to attend study meetings at Pre- and Post-Challenge sessions, it was decided that meeting the ≥25% increase by either pre- or post-challenge sessions would constitute compliance in the experimental group. Since the baseline oleic and palmitoleic acid blood levels in the participants were very high, it was determined to be unlikely that the DSs provided for the control group would increase the blood levels sufficiently. As a result, since the control group was asked to no longer consume any DSs with omega-3 fatty acids for the duration of the study (as criterion for inclusion), only those in the control group who did not increase omega-3 blood levels from baseline to either pre- or post-challenge assessments were defined as control compliers and were used in the APP analysis. The final sample for the APP analysis was *n* = 251 and was comprised of 118 participants from the control group and 133 individuals from the experimental group. No statistically significant differences in any of the main or secondary cognitive outcomes were identified using the APP analysis with the same cognitive tests. Similar to the ITT analysis, for some of the cognitive tests, a statistically significant main effect of time was demonstrated. These data can be found in Appendix C, Table A3.

## 4. Discussion

These data did not demonstrate that supplemental krill-based, omega-3 fatty acids significantly improved performance on a wide array of cognitive assessments in comparison with control in young US Army officers during a 17- or 19-week training period. This study was challenging for the study participants who were expected to consume 8 capsules per day while engaged in intensive military training, which had a significant impact on their future career. Moreover, some participants may have found it challenging to consume DSs while engaging in field exercises. In addition, a number of participants shared a living space or lived in the same building as other participants and all the participants were in platoons with other participants. Due to the close living and working arrangements, participants were more likely to compare their study experiences and possibly their DSs. The scientific team was told on multiple occasions that some participants opened their DSs to try to determine whether they were part of the experimental or control group. Knowing these facts, the study design called for both ITT and APP analyses. However, the APP analysis, which required at least a 25% increase in blood levels of omega-3 levels, also did not demonstrate any statistically significant differences between the experimental and control groups, even with the relatively high dose of omega-3 supplementation.

Most RCTs that have addressed omega-3 fatty acid supplements and cognitive function have focused on young children or the elderly as study participants, with the research focused on cognitive development or cognitive decline [3]. One RCT compared omega-3 supplementation in healthy older adults sourced from krill and sardines to placebo and found that both produced increased oxyhemoglobin concentrations (as measured by near-infrared spectroscopy) during a task of working memory [37]. Moreover, compared to placebo, participants assigned to krill oil supplement showed reduced P300 latency on the working memory task and increased oxyhemoglobin on another task involving mental calculation. While these physiologic markers of brain activity appeared to improve with supplementation, the authors did not report changes in behavioral performance. Very few studies have evaluated cognitive functioning in healthy young adults [3,38]. These studies have found mixed results [2,3,39]. Cook et al. [40] assessed omega-3 fatty acid status among 299 healthy young women (aged 18–35 years) and reported that women with a lower omega-3 index had lower attention scores on a series of cognitive measures. Another study involving a smaller sample of healthy adults (*n* = 13) comprised primarily of women (*n* = 9), found EPA supplementation to reduce reaction times on a measure of inhibition [34]. Discrepancies between these studies and the present investigation might be due to gender differences in absorption and/or response to omega-3. In a study with a sample more similar to our investigation, Dretsch et al. [41] found no improvement in a number of measures of neurocognition but did find a decrease in daytime sleepiness among deployed soldiers. This finding is similar to another RCT with healthy older adults that did not find benefit on measures of cognitive performance with omega-3 supplementation, despite good adherence to a 1 g/d intervention [42]. Finally, Stonehouse et al. [35] found that omega-3 supplementation reduced reaction time on a test of working memory in adult men; however, these individuals were pre-selected based on very low dietary *n − 3* consumption at baseline. As such, it is possible that a cognitive benefit from supplementation in otherwise healthy adults may be predicated on pre-existing deficiency.

However, with the poor retention rate of only 44.1% and the questionable compliance among the individuals who completed the study, we feel that the study also may have been underpowered to test the null hypotheses, even though an interim power analysis was performed in which 50% attrition was assumed. In addition, the rumors about participants opening the DS capsules and sharing the experimental capsules also may have contributed to the low blood levels of omega-3s.

## 5. Conclusions

Daily supplementation with omega-3 fatty acids compared to control was not associated with improvement in any measured cognitive tests among young adult military officers. Other studies of highly unsaturated omega-3 fatty acid supplementation have reported in an improvement in specific tests of memory with sex differences demonstrated. The lack of a significant effect in this study may reflect the stressful situation represented by military officer training. However, the poor retention rate and questionable compliance coupled with group living conditions possibly leading to DS mishandling, leads to concerns about the overall study power to test the null hypotheses. Thus, we cannot definitely conclude that there was no effect. Given the US Army Training and Doctrine Command’s (TRADOC) recent emphasis on holistic health and fitness, more research is needed to inform overall nutritional requirements to optimize soldier performance.

## Figures and Tables

**Table 1:** Krill Oil Fatty Acid Composition.

Fatty Acid	Percent	Fatty Acid	Percent
C14:0	3.1	C20:4n − 6	0.2
C16:0	16.8	C22:4n − 6	<0.1
C18:0	0.7	C18:3n − 3	1.2
C20:0	<0.1	C18:4n − 3	2.1
C22:0	<0.1	C20:3n − 3	0.1
C16:1n − 7	1.7	C20:4n − 3	0.3
C18:1(n−) + (n − 7) + (n − 5)	8.3	C20:5n − 3	19.0
C20:1(n − 9) + (n − 7)	0.4	C21:5n − 3	0.5
C22:1(n − 11) + (n − 9) + (n − 7)	0.8	C22:5n − 3	0.3
C24:1n − 9	0.1	C22:6n − 3	10.0
16:2n − 4	0.1		
16:3n − 4	<0.1	SFA	20.5
C18:2n − 6	0.9	MEFA	11.1
C18:3n − 6	0.1	PUFA (n − 6)	1.2
C20:2n − 6	<0.1	PUFA (n − 3)	33.4
C20:3n − 6	<0.1	Total PUFA	34.7
Total Fatty Acids			67.0

**Table 2:** Macadamia Nut Oil Composition.

Fatty Acids		Percentage of Total Fatty Acids
Lauric acid	(C12:0)	1.0%
Myristic acid	(C14:0)	1.5%
Palmitic acid	(C16:0)	10.0%
Palmitoleic acid	(C16:1)	24.0%
Stearic acid	(C18:0)	4.0%
Oleic acid	(C18:1)	67.0%
Linoleic acid	(C18:2)	4.0%
Linolenic acid	(C18:3)	0.5%
Arachidic acid	(C20:0)	3.0%
Eicosenoic acid	(C20:1)	3.0%
Behenic acid	(C22:0)	1.0%
Erucic acid	(C22:1)	1.0%
Lignoceric acid	(C24:0)	0.5%

**Table 3:** Baseline Characteristics of Randomized Participants, Overall and by Treatment Group *n* (%) or median (IQR) IQR = Interquartile Range (Q1, Q3).

Characteristic	Overall *n* = 555	Omega-3 *n* = 276 (49.7%)	Placebo *n* = 279 (50.5%)	*p*-Value *
Male	546 (98.6)	274 (99.3)	273 (97.9)	0.1110
Age (years)				0.8602
≤21	27 (4.9)	14 (5.1)	13 (4.7)	
22	233 (42.1)	111 (40.4)	123 (44.1)	
23	138 (25.0)	68 (24.7)	70 (25.1)	
24–28	119 (21.5)	62 (22.6)	57 (20.4)	
≥29	36 (6.5)	20 (7.3)	16 (5.7)	
Race/Ethnicity				0.6650
Non-Hispanic White	440 (79.3)	216 (78.3)	224 (80.3)	
Non-Hispanic Black/Africa American	39 (7.0)	17 (6.2)	22 (7.9)	
Hispanic	46 (8.3)	27 (9.8)	19 (6.8)	
Non-hispanic Asian	25 (4.5)	13 (4.7)	12 (4.3)	
Other	5 (0.9)	3 (1.1)	2 (0.7)	
Military Service				
Commissioning Source				0.9961
USMA	135 (24.3)	68 (24.6)	67 (24.0)	
ROTC	337 (60.7)	167 (60.5)	170 (60.9)	
OCS	82 (14.8)	41 (14.9)	41 (14.7)	
DC	1 (0.2)	0	1 (0.4)	
Post-graduation Destination				0.9972
IBCT-ABN (Infantry Airborne)	268 (48.3)	133 (48.2)	135 (48.4)	
IBCT-Light Infantry (not Airborne)	169 (30.5)	85 (30.8)	84 (30.1)	
ABCT-Armored	31 (5.6)	15 (5.4)	16 (5.7)	
SCBT-Stryker	87 (15.7)	43 (15.6)	44 (15.8)	
Education				0.8521
Bachelor’s Degree	532 (95.9)	265 (96.0)	267 (95.7)	
Master’s Degree or PhD	23 (4.1)	11 (4.0)	12 (4.3)	
Marital Status				0.9466
Married	100 (18.0)	49 (17.8)	51 (18.3)	
Never married	442 (79.6)	220 (79.7)	222( 79.6)	
Cohabitating/Sep/Divorced	13 (2.3)	7 (2.5)	6 (2.2)	
Number of people in household				0.7888
1	141 (26.0)	76 (27.5)	68 (24.4)	
2	152 (27.4)	78 (28.3)	74 (26.5)	
3	125 (22.5)	61 (22.1)	64 (22.9)	
4	115 (20.7)	52 (18.8)	63 (22.6)	
5+	19 (3.4)	9 (3.3)	10 (3.6)	
Self-reported Total household income during last 12 months				0.3258
Less than $10,000	48 (8.7)	23 (8.4)	25 (9.0)	
$10,000–$19,999	45 (8.1)	26 (9.5)	19 (6.8)	
$20,000–$29,000	52 (9.4)	22 (8.0)	30 (10.8)	
$30,000–$39,000	112 (20.3)	62 (22.6)	50 (18.0)	
$40,000–$49,000	95 (17.2)	40 (14.6)	55 (19.8)	
$50,000–$59,000	60 (10.9)	25 (9.1)	35 (12.6)	
$60,000–$74,999	41 (7.4)	21 (7.6)	20 (7.2)	
$75,000 or more	60 (10.9)	34 (12.4)	26 (9.4)	
Prefer not to answer	40 (7.2)	22 (8.0)	18 (6.5)	

* *p*-value from Chi Square Test or Wilcoxon Rank Sum Test.

**Table 4:** Estimated mean usual nutrient intake [27] of RRIPP-3 participants at baseline based on 24-h dietary recall and a 30-day food frequency questionnaire plus intake of DSs that were collected during the baseline visit.

	Nutrients from Diet ^1^	Nutrients from DSs ^2^	Total Nutrients	Experimental Group (*n* = 256)	Control Group (*n* = 261)	MDRIs ^3,4,5^
Mean (SD) or Median (Q1, Q3) *n* = 517						
Energy (kcal/day)	3021.6	132.0	3105.8	3102.4	3109.1	3400
	(1211.5)	(100.0, 232.5)	(1244.8)	(1203.8)	(1286.0)	
Protein (g/day)	152.7	34.3	165.1	159.2	170.8	102
	(69.9)	(22.3)	(75.7)	(68.3)	(82.0)	(68–136)
Carbohydrate (g/day)	322.6	5.0	328.7	327.9	329.5	510
	(152.9)	(3.0, 10.5)	(157.4)	(160.2)	(154.9)	(340–680)
Total Fat (g/day)	125.4	3.2	126.6	129.6	123.7	<113
	(61.8)	(4.5)	(62.2)	(62.5)	(61.8)	
Linoleic Acid	25.6	12.0	25.7	26.9	24.5	17
(g/day)	(15.4)	(2.8)	(15.4)	(16.1)	(14.8)	
α-Linolenic Acid	2.3	0.9	2.4	2.4	2.3	1.6
(g/day)	(1.6)	(0.6)	(1.6)	(1.6)	(1.6)	
EPA (mg/day)	13.0	14.0	12.0	12.0	16.0	ND
	(7.0, 28.0)	(7.0, 32.0)	(7.0, 29.0)	(7.0, 29.0)	(8.0, 33.0)	
DHA (mg/day)	77.0	82.0	85.5	85.5	81.0	ND
	(28.0, 131.0)	(32.0, 150.0)	(31.0, 133.0)	(31.0, 133.0)	(33.0, 162.0)	
Dietary Fiber(g/day)	25.6	2.5	26.0	26.5	25.5	34
	(15.2)	(2.9)	(15.3)	(15.6)	(15.0)	
Vitamin A (ug/RAE/day) ^6^	1188.0	3805.7	1184.6	1264.6	1826.4	900
	(906.8)	(2914.8)	(669.6, 2391.9)	(681.2, 2431.2)	(1775.5)	
Vitamin D (ug/day)	7.2	20.0	8.4	8.2	8.6	15
	(4.4, 12.2)	(10.0, 25.0)	(4.7, 15.7)	(4.5, 15.3)	(4.9, 16.0)	
Vitamin E as alpha tocopherol (mg/day)	11.6	27.0	14.6	15.6	13.7	15
	(7.8, 20.5)	(20.3, 36.2)	(8.6, 31.2)	(9.1, 32.3)	(8.1, 29.7)	
Vitamin K (ug/day)	104.8	49.7	113.7	116.5	109.8	120
	(59.2, 205.2)	(32.0)	(62.3, 207.2)	(65.7, 206.0)	(59.2, 212.4)	
Thiamin (mg/day)	2.7	3.0	2.5	2.5	2.5	1.2
	(2.0)	(1.4, 25.0)	(1.8, 3.7)	(1.8, 3.8)	(1.8, 3.6)	
Riboflavin (mg/day)	3.6	3.4	3.4	3.4	3.4	1.3
	(2.3)	(1.7, 25.0)	(2.3, 5.1)	(2.3, 4.9)	(2.4, 5.2)	
Niacin (mg NE/day) ^7^	52.6	32.8	60.3	61.2	59.3	14
	(30.2)	(26.6)	(36.7)	(37.6)	(35.9)	
Vitamin B6 (mg/day)	4.4	4.0	4.4	4.4	4.4	1.3
	(3.1)	(2.0, 10.0)	(2.9, 6.9)	(2.8, 6.9)	(3.0, 6.8)	
Vitamin B12 (ug/day)	8.5	25.0	11.8	11.8	11.7	2.4
	(5.0, 15.0)	(12.0, 50.9)	(6.0, 26.6)	(6.5, 27.4)	(5.9, 26.1)	
Folate (DFE) (ug/day)	809.6	376.3	896.5	888.9	904.0	400
	(526.8)	(178.9)	(561.7)	(494.7)	(621.3)	
Vitamin C (mg/day)	93.2	100.0	118.7	119.2	115.8	90
	(42.6, 160.9)	(60.0, 300.0)	(58.5, 232.0)	(57.3, 227.0)	(59.1, 233.5)	
Calcium (mg/day)	1528.9	200.0	1601.3	1544.0	1657.6	1000
	(1000.6)	(96.7, 259.9)	(1029)	(960.6)	(1090.9)	
Iron (mg/day)	24.9	2.5	25.7	25.4	26.1	8
	(17.3)	(0.6, 10.0)	(18.3)	(16.2)	(20.1)	
Magnesium (mg/day)	519.4	100.0	542.2	543.3	541.2	420
	(339.9)	(50.0, 140.0)	(352.9)	(318.9)	(383.9)	
Phosphorus (mg/day)	2366.5	79.5	2377.4	2329	2424.9	700
	(1187.3)	(38.0, 130.0)	(1194.7)	(1130.5)	(1254.8)	
Potassium (mg/day)	4234.6	200.0	4310.0	4196.4	4421.4	4700
	(2143.9)	(115.0, 320.0)	(2172.5)	(1954.7)	(2365.3)	
Selenium (ug/day)	212.7	117.8	229.6	228.2	231.0	55
	(109.2)	(78.0)	(120.0)	(116.5)	(123.6)	
Sodium (mg/day)	5854.1	225.9	5941.1	5713.2	6164.6	<2300
	(2622.8)	(213.1)	(2648.6)	(2308.6)	(2930.7)	
Zinc (mg/day)	19.9	13.7	22.5	22.6	22.4	11
	(13.1)	(9.9)	(14.8)	(13.9)	(15.7)	

^1^ Diet determined by self-report at study initiation based on the United States Department of Agriculture (USDA) Automated Multiple-Pass Method (AMPM) for assessing dietary intake, which is part of the United States National Health and Nutrition Examination Survey (NHANES) [25]. ^2^ Dietary Supplement Intake based on self-report based on the Centers for Disease Control and Prevention (CDC) computerized dietary assessment intake component of the NHANES [36]. ^3^ While the study sample included 4 females, these data are limited to the male sample. ^4^ MDRI values were established by Army Regulation 40–25, OPNAVINST 10110.1/MCO 10110.49, AFI 44-141 effective 3 February 2017 as nutrition standards for military feeding and operational rations based on the Food and Nutrition Board, National Academy of Sciences Dietary Reference Intake recommendations for American adult males, where US DRIs are available. Values in the table represent the MDRIs for men. ^5^ Range represents MDRI for light activity, moderate activity, heavy activity and exceptionally heavy activity. ^6^ RAE = retinol activity equivalents; ^7^ NE = niacin equivalents.

## Data Availability

The data presented in this study are available on request from the corresponding author. The data are not publicly available because further release of the data has not been approved by the US Department of the Army.

## Appendix A

**Table A1:** Baseline health and lifestyle characteristics of RRIPP-3 study participants overall and by treatment group.

Characteristic	Overall	Omega-3	Placebo	*p*-Value *
BMI	26.9 ± 2.6	26.9 ± 2.6	27.0 ± 2.6	0.6470
Height (cm) ^†^	177.4 ± 7.0	177.6 ± 6.6	177.2 ± 7.3	0.5369
Weight (lbs) ^†^	186.8 ± 23.1	186.8 ± 22.7	186.7 ± 23.4	0.9673
Self-reported General Health				0.9573
Excellent	256 (46.1)	125 (45.3)	131 (47.0)	
Very good	253 (45.6)	129 (46.7)	124 (44.4)	
Good	44 (7.9)	21 (7.6)	23 (8.2)	
Fair/poor	2 (0.4)	1 (0.4)	1 (0.4)	
Past 30-day drinker (yes)	495 (89.2)	248 (89.9)	247 (88.5)	0.6153
Use of Tobacco Products				
Ever smoked > 100 cigarettes				0.6105
Yes	41 (7.4)	18 (6.5)	23 (8.2)	
No	491 (88.5)	245 (88.8)	246 (88.2)	
Refused/Don’t know	23 (4.1)	13 (4.7)	10 (3.6)	
Now smoke cigarettes	0	0	0	
Ever used chewing tobacco/snuff	235 (42.3)	117 (42.4)	118 (42.3)	0.4083
Chewing tobacco Past 12 months				0.3667
About every day	43 (18.3)	22 (18.8)	21 (17.8)	
3–6 days/week	31 (13.2)	16 (13.7)	15 (12.7)	
1–2 days/week	27 (11.5)	18 (15.4)	9 (7.6)	
1–3 days/month	35 (14.9)	17 (14.5)	18 (15.3)	
Less than once/month; Not used Past 12 months	99 (42.1)	44 (37.6)	55 (46.6)	
Ever user electronic or smoking nicotine delivery product (Ex. E-cig)	78 (14.1)	43 (15.6)	35 (12.5)	0.3037
Ever user caffeinated smokeless tobacco	67 (12.1)	27 (9.8)	40 (14.3)	0.0996
Moderate Physical Activity Frequency				0.3582
Every day	219 (39.5)	112 (40.6)	107 (38.4)	
5–6 days/week	167 (30.1)	83 (30.1)	84 (30.1)	
3–4 days/week	111 (20)	49 (17.8)	62 (22.2)	
1–2 days/week	52 (9.4)	27 (9.8)	25 (9.0)	
1–3 days/month or never	6 (1.1)	5 (1.8)	1 (.4)	
Vigorous Physical Activity Frequency				0.2321
Every day	90 (16.2)	48 (17.4)	42 (15.1)	
5–6 days/week	251 (45.2)	128 (46.4)	123 (44.1)	
3–4 days/week	161 (29)	69 (25.0)	92 (33.0)	
1–2 days/week	44 (7.9)	25 (9.1)	19 (6.8)	
1–3 days/month or never	9 (1.6)	6 (2.2)	3 (1.1)	
Baseline Medical Conditions				
Cough	30 (5.4)	17 (6.2)	13 (4.7)	0.4346
Runny Nose	47 (8.5)	19 (6.9)	28 (10.0)	0.1824
Sneezing	36 (6.5)	17 (6.2)	19 (6.8)	0.7557
Asthma	16 (2.8)	10 (3.6)	6 (2.2)	0.2999
Allergies	78 (14.1)	37 (13.4)	41 (14.7)	0.6621
Chronic pain	15 (2.7)	6 (2.2)	9 (3.2)	0.6023
Pain that lasts more than 24 hours	51 (9.2)	27 (9.8)	24 (8.6)	0.6303
Concussions	71 (12.8)	33 (12.0)	38 (13.6)	0.5574
Gas	50 (9.0)	34 (12.3)	16 (5.7)	0.0068
Any skin condition	33 (6.0)	17 (6.2)	16 (5.7)	0.8325

* *p*-values from Chi Square Tests of Association, Fisher’s Exact Test or Student’s test; ^†^ Height and weight were measured 3 times, then averaged; BMI = Body Mass Index.

## Appendix B

**Table A2:** Fatty Acid Blood Levels for study participants in the treatment and placebo groups at baseline, pre-challenge and post challenge as in the As-Per-Protocol Analysis.

	Baseline		Pre-Challenge		Post-Challenge	
Fatty Acid	Placebo (*n* = 261)	Treatment (*n* = 257)	Placebo (*n* = 86)	Treatment (*n* = 108)	Placebo (*n* = 142)	Treatment (*n* = 171)
14_0	1.28 (0.59)	1.29 (0.57)	1.44 (0.48)	1.5 (0.53)	1.19 (0.61)	1.14 (0.62)
16_0	26.07 (3.65)	25.59 (4.6)	26.69 (2.69)	26.61 (2.57)	25.76 (3.22)	26.07 (2.97)
18_0	14.55 (2.93)	14.27 (1.81)	13.83 (1.59)	13.9 (1.37)	14.05 (1.54)	14.06 (1.57)
18_1n7	1.49 (1.28)	1.35 (0.29)	1.28 (0.22)	1.23 (0.19)	1.66 (2.37)	1.44 (1.38)
18_1n9	15.1 (2.99)	15.39 (2.68)	16.9 (2.91)	16.08 (2.63)	15.19 (3.52)	14.86 (2.49)
18_2n6	17.83 (2.57)	18.12 (2.55)	18.03 (2.7)	17.87 (2.26)	18.93 (3)	18.89 (2.74)
18_3n6	0.22 (0.29)	0.2 (0.13)	0.25 (0.14)	0.24 (0.12)	0.22 (0.12)	0.2 (0.12)
20_0	0.66 (0.32)	0.64 (0.19)	0.5 (0.1)	0.49 (0.1)	0.56 (0.15)	0.56 (0.21)
20_1n9	0.43 (0.46)	0.41 (0.41)	0.22 (0.07)	0.22 (0.07)	0.24 (0.09)	0.22 (0.08)
20_2n6	0.23 (0.1)	0.22 (0.09)	0.39 (0.26)	0.4 (0.24)	0.29 (0.18)	0.3 (0.19)
20_3n6	1.04 (0.32)	1.02 (0.31)	1.19 (0.33)	1.07 (0.33)	1.13 (0.3)	1.05 (0.29)
20_4n6	7.84 (1.55)	7.99 (1.61)	7.02 (1.33)	6.7 (1.18)	7.95 (1.5)	7.34 (1.4)
22_0	1.9 (0.41)	1.89 (0.43)	1.76 (0.35)	1.8 (0.27)	1.91 (0.36)	1.86 (0.37)
22_1n9	0.47 (0.39)	0.51 (0.53)	0.2 (0.24)	0.2 (0.23)	0.25 (0.45)	0.25 (0.34)
22_4n6	1.34 (0.41)	1.38 (0.45)	1.26 (0.59)	0.99 (0.68)	1.2 (0.32)	0.9 (0.27)
22_5n3	0.52 (0.3)	0.53 (0.31)	0.65 (0.17)	0.9 (0.24)	0.7 (0.18)	0.95 (0.22)
22_5n6	0.38 (0.24)	0.38 (0.2)	0.25 (0.41)	0.28 (0.79)	0.31 (0.49)	0.25 (0.39)
24_0	3.42 (2.33)	3.58 (2.84)	2.85 (0.5)	2.97 (0.46)	3.15 (0.49)	3.18 (0.45)
24_1n9	2.45 (0.64)	2.43 (0.58)	2.18 (0.48)	2.58 (2.97)	2.48 (1.11)	2.53 (0.92)
22_6n3	1.4 (0.51)	1.41 (0.4)	1.21 (0.47)	1.8 (0.55)	1.27 (0.4)	1.96 (0.6)
20_5n3	0.25 (0.19)	0.25 (0.16)	0.23 (0.2)	0.8 (0.64)	0.24 (0.26)	0.77 (0.64)
18_3n3	0.43 (0.27)	0.43 (0.27)	0.53 (0.4)	0.49 (0.27)	0.58 (0.71)	0.47 (0.37)

## Appendix C

**Table A3:** Results for the Cognitive Assessments using the As-Per-Protocol (APP) Analysis Approach where the participants in the experimental group demonstrated a ≥25% increase from baseline in *n − 3* blood levels and the participants in the control group demonstrated no increase from baseline in *n − 3* blood levels.

		Time of Assessment				
Test	Treatment	Baseline	Pre-Challenge	Post-Challenge	*p*-Value *	*p*-Value (Time) *
		LS Mean * (SE)	LS Mean (SE)	LS Mean (SE)		
Stroop	Experimental	51.0 (1.7)	60.4 (1.8)	66.2 (1.8)	0.7120	<0.0001
	Control	55.8 (1.8)	64.1 (2.0)	70.6 (2.0)		
Digit Symbol	Experimental	73.9 (1.5)	77.4 (1.7)	75.7 (1.8)	0.6743	0.2450
	Control	75.1 (1.6)	77.9 (2.0)	75.3 (1.9)		
Connor-Davidson	Experimental	81.0 (0.9)	83.6 (1.0)	82.8 (1.1)	0.8728	0.5168
	Control	83.0 (1.0)	84.0 (1.1)	83.5 (1.3)		
PROMIS: Fatigue Scale	Experimental	15.6 (0.5)	16.4 (0.6)	19.8 (0.6)	0.2574	<0.0001
	Control	7.0 (0.6)	18.0 (0.6)	20.5 (0.6)		
PROMIS: Sleep-related Impairment Scale	Experimental Control	17.2 (0.5) 18.3 (0.5)	17.0 (0.5) 18.1 (0.5)	9.5 (0.5) 20.4 (0.5)	0.8927	<0.0001
PROMIS: Applied Cognition Scale	Experimental	32.1 (0.6)	32.8 (0.6)	30.0 (0.7)	0.4962	0.0004
	Control	31.7 (0.7)	31.4 (0.6)	29.2 (0.7)		
Balloon Analogue Risk Task (BART)	Experimental	20.2 (0.6)	23.6 (0.8)	27.0 (0.7)	0.6766	<0.0001
	Control	19.4 (0.7)	23.2 (0.8)	26.2 (0.8)		
Grammatical Reasoning Test	Experimental	26.8 (0.4)	27.3 (0.4)	27.1 (0.3)	0.1552	0.1104
	Control	27.7 (0.4)	27.8 (0.4)	27.1 (0.4)		
Four-Choice Serial Reaction Time Test	Experimental	479.3 (1.5)	475.5 (1.7)	469.7 (1.9)	0.5922	0.0003
	Control	479.0 (1.5)	476.3 (1.8)	469.1 (2.0)		
Spatial Working Memory Test	Experimental	21.9 (0.5)	22.9 (0.6)	22.9 (0.6)	0.5010	0.4208
	Control	22.4 (0.7)	22.95 (0.6)	22.4 (0.6)		
Spielberger State Anxiety Inventory	Experimental Control	31.7 (0.8) 32.5(0.8)	30.6 (0.8) 32.0 (0.9)	31.8 (0.8) 33.7 (0.9)	0.6262	0.0279
POMS: Composed/ Anxious Scale	Experimental	27.5 (0.5)	27.8 (0.5)	27.1 (0.5)	0.8986	0.2151
	Control	26.6 (0.6)	27.2 (0.6)	26.6 (0.6)		
POMS: Agreeable/Hostile Scale	Experimental	28.7 (0.4)	28.0 (0.5)	25.7 (0.6)	0.8798	<0.0001
	Control	28.2 (0.4)	27.2 (0.5)	25.0 (0.6)		
POMS: Elated/Depressed Scale	Experimental	27.1 (0.5)	27.1 (0.6)	25.5 (0.6)	0.1038	0.3171
	Control	26.5 (0.5)	25.9 (0.6)	25.4 (0.6)		
POMS: Confident/Unsure Scale	Experimental	26.9 (0.5)	28.3 (0.5)	27.3 (0.5)	0.6838	0.0057
	Control	26.2 (0.5)	27.1 (0.5)	25.9 (0.6)		
POMS: Energetic/Tired Scale	Experimental	24.9 (0.6)	24.7 (0.6)	20.4 (0.7)	0.2082	<0.0001
	Control	23.6 (0.6)	23.3 (0.7)	20.2 (0.7)		
POMS: Clearheaded/Confused Scale	Experimental	29.7 (0.4)	30.1 (0.5)	28.5 (0.5)	0.9154	0.0004
	Control	29.0 (0.5)	28.7 (0.5)	27.0 (0.6)		

* *p*-values from mixed modeling for repeated measures; LS Mean = Least Square Mean; SE = Standard Error.
